# Dilated Cardiomyopathy in Children and Adults: What is New?

**DOI:** 10.1100/tsw.2008.105

**Published:** 2008-08-06

**Authors:** Galal E. Nagib Elkilany, Mustafa A. AL-Qbandi, Khaled A. Sayed, Ibrahim Kabbash

**Affiliations:** ^1^ Adult Cardiology Department, Echocardiography Laboratory, Chest Hospital, Safat, Kuwait; ^2^ Consultant Cardiologist, Cardiology Department, Tanta University, Egypt; ^3^ Pediatric Cardiology Department, Chest Hospital, Safat, Kuwait; ^4^ Public Health Department, Tanta Faculty of Medicine, Egypt

**Keywords:** dilated cardiomyopathy, echocardiography, strain

## Abstract

Dilated cardiomyopathy (DCM) is the most common form of cardiomyopathy and cause of cardiac transplantation in children and young adults; mortality is high among this patient population. However, mortality, clinical course, and illustrative echocardiographic data of DCM in children and adults are not well established. Our objective was to provide a research article of detailed descriptions of the incidence, causes, outcomes, related risk factors, and new echocardiographic criteria of risk of death from DCM. Our results showed that independent risk factors at DCM diagnosis for subsequent death or transplantation in children cohorts were older age, congestive heart failure, lower left ventricular ejection fraction (EF ≤ 25%), low global strain, significant mitral valve incompetence, pulmonary hypertension, diastolic dysfunction, right ventricular involvement, and cause of DCM (*p* < 0.001 for all). In adults, low ejection fraction (<30–35%), global peak systolic strain <-7.6%, increased EDV, ESV, LBBB, diastolic dysfunction, and left ventricle dyssynchrony were the main independent risk factors for major cardiac events and need for CRT or transplantation (*p* < 0.001 for all). Our conclusions were that in children and adults, DCM is a diverse disorder with outcomes that depend largely on cause, age, heart failure status at presentation, and echocardiographic parameters of the heart (systolic and diastolic function of left ventricle, pulmonary artery pressure, global strain, and valvular function of the mitral valve). This study will present new findings in the diagnostic area.

## INTRODUCTION

Dilated cardiomyopathy (DCM) is the most common form of cardiomyopathy and cause of cardiac resynchronization therapy (CRT), cardiac transplantation, and mortality in children and adults. However, the epidemiology, detailed echocardiographic criteria of risk, and clinical course of DCM in children as well as in adults are not well established.

Cardiomyopathies are heart muscle disorders that affect ventricular systolic function, diastolic function, or both. Despite long-standing interest in these high-impact disorders, the demographics and underlying causes have been difficult to ascertain, particularly in children.

DCM is a myocardial disorder characterized by a dilated left ventricular (LV) chamber and systolic dysfunction that commonly results in congestive heart failure (CHF)[1,2]. In some cases, right ventricular dysfunction (RVD) is also noted and may add to the clinical severity of disease[3,4]. The estimated cost of caring for patients with this disorder is $4–10 billion annually in the U.S. alone[5,6]. In adults, the incidence of DCM has been reported to be 5.5 cases/100,000 population/year, with a prevalence of 36 cases/100,000 population[7,8]. The underlying cause in adults[2] is usually coronary artery disease (CAD), but other causes are also seen, including inflammatory heart disease, myocardial toxins, and genetic defects[9,10]. Approximately 30–35% of patients are reported to have a genetic form of DCM[11,12,13,14]. Infants and older children, however, appear to have a wider spectrum of causes[9,15,16,17], although identifying these causes has been difficult.

Relatively little information on the incidence of cardiomyopathies in childhood has been published[18,19,20]. Arola et al.[20] reported an incidence of DCM of 0.34 cases/100,000 children/year and a prevalence of 2.6 cases/100,000 children in Finland, a racially homogeneous population. A large percentage of cases occurred in infants (<1 year of age; 3.8/100,000 cases/year). Recently, the Pediatric Cardiomyopathy Registry (PCMR) reported the incidence of pediatric cardiomyopathy in two regions of the U.S.: New England and the central Southwest[21]. A total of 467 cases of childhood cardiomyopathy was reported, yielding an annual incidence of 1.13/100,000 infants and children overall, with differences by race, sex, and region. These data are supported by similar findings from Australia[22]. The PCMR report defines the overall incidence of all forms of childhood cardiomyopathy, but has limited details classified by the World Health Organization as (1) DCM, (2) hypertrophic cardiomyopathy, (3) restrictive cardiomyopathy, and (4) arrhythmogenic RV dysplasia-cardiomyopathy[1]. Most patients have “pure” forms of these disorders that fulfill strict diagnostic criteria, although some have overlapping disorders, with mixed forms of disease regarding the causes, risks, and outcomes of the specific forms of cardiomyopathy. However, more detailed information focusing on particular forms of cardiomyopathy is required in order for clinicians to understand the clinical disorders of individual patients.

The current study provides the most up-to-date estimates of the incidence of DCM in children and adults living in Kuwait and Egypt, as well as a detailed description of the causes, outcomes, echocardiographic findings, and related risk factors for DCM in children and adults.

## METHODS

### Design and Setting

This was a longitudinal study based on a population-based, prospective cohort of children and adults diagnosed as having DCM since February 1, 2007 (at two pediatric cardiac centers and one adult tertiary center), and a retrospectively collected cohort of patients seen primarily at large tertiary care centers in Kuwait and Egypt and who had diagnoses between January 1, 1996 and February 29, 2008.

### Participants

A total of 195 cases (90 children and 105 adults) from Kuwait and Egypt, diagnosed as having DCM at younger than 18 years for the children’s group and from 20 to 78 years for the adult group (mean of 48 ± 12 years) were involved in the study. Primary DCM was determined by strict medical history, echocardiography, and pathologic criteria for selected patients.

## Eligibility Criteria

All patients with cardiomyopathy were identified by clinical presentation to a pediatric cardiologist (for children) and adult cardiologist (for adults) with signs and symptoms of heart failure, sudden death or aborted sudden death, or evaluation for possible cardiomyopathy because of familial inheritance. In addition, autopsy reports were evaluated in a retrospective case review. Sudden death was captured by review of the cardiology and pathology medical records. A variety of diagnostic exclusion criteria[23] were used, including endocrine disorders or immunologic diseases known to cause heart muscle disease, and inflammation caused by human immunodeficiency virus (HIV) infection (or birth to an HIV-positive mother) or by Kawasaki disease.

A strict quantitative echocardiographic criteria of LV dilation, systolic dysfunction (low EF), EDV (end-diastolic volume), ESV (end-systolic volume), severity of mitral valve incompetence, RV involvement, pulmonary hypertension, LV dyssynchrony (by tissue Doppler imaging, tissue synchronization imaging, strain, and four-dimensional LV volume), LBBB (left bundle branch block), diastolic dysfunction, and GLPSS (global peak systolic strain) are met in 70% of children and 100% of adults.

This analysis focuses on pure DCM, defined as the presence of DCM at diagnosis, excluding any additional overlapping cardiac phenotype; cases of mixed functional DCM, including a combination of DCM with hypertrophic, restrictive, and arrhythmogenic RV cardiomyopathy.

## Data Collection

Supplemental information on clinical history, procedures, and outcomes is obtained annually for all patients, and information on family history, results of laboratory studies, and therapies administered is additionally collected for retrospective cohort patients.

## Statistical Methods

Descriptive statistics are presented as percentages or means and standard deviations, with skewed continuous data summarized as medians and interquartile ranges. The distributions of categorical variables were compared using the Monte Carlo exact test, except for comparisons by cause, for which the $\chi 2$ statistic was used. Two groups of normally distributed variables were compared using the t test, and analysis of variance was used to compare more than two groups. Skewed data were analyzed using the Wilcoxon rank-sum test and the Kruskal-Wallis test. The Mantel-Haenszel test for linear trend was used to examine age at diagnosis of cardiomyopathy grouped categorically by cause. Correlation coefficient (r) was used for comparison of GLPSS values vs. ejection fraction in DCM patients.

LV end-diastolic and end-systolic dimensions and volumes (LVEDD, LVEDV, LVESD, LVESV), posterior wall thickness, septal thickness, and mass were measured. Fractional shortening (and ejection fraction) is a measure of LV contractility and is defined by the ratio of the difference between the end-diastolic dimension (LVEDD) and end-systolic dimension (LVESD) to the LVEDD, expressed as fractional shortening = (LVEDD – LVESD)/LVEDD × 100. Quantitative RV structure and function data were collected in M-Mode (tricuspid annular plane systolic excursion), two-dimensional (systolic shrinkage area), and TDI (tissue Doppler imaging) by measurements of absolute value of systolic velocity of tricuspid annulus lateral systolic velocity. Pulmonary artery systolic pressure was measured through tricuspid regurgitation peak systolic velocity (continuous wave Doppler) and inferior vena cava diameter and collapsibility index. The severity of mitral valve incompetence was currently evaluated through color Doppler (absolute regurgitant jet area, ratio of jet area to left atrial area, vena contracta, and effective regurgitant orifice area) with two- and three-dimensional echocardiography. Diastolic dysfunction in the present study was measured by standard mitral flow (E/A velocity ratio and E deceleration time), mitral annular velocity (TDE), and pulmonary venous flow pattern.

## Assessment of GLPSS in DCM (Automated Function Imaging)

The automated function imaging (AFI) algorithm noninvasively tracks and analyzes peak systolic strain based on two-dimensional strain. Digital loops were acquired from apical two-, three-, and four-chamber views and a line was traced along the LV endocardium. Around this line, the software selected natural acoustic markers moving with the tissues' automatic frame by frame tracking of these markers during the heart cycle, yielding a measure of contractility along the selected region of interest. GLPSS were calculated for the entire U-shaped length of the LV myocardium (basal, mid, and apical segments of two opposite walls in each view) in 105 consecutive patients with DCM (70 adults, 35 children) (Fig. 1).

Outcome measures were recovery (improvement), death, CRT, and cardiac transplantation.

Because of varying amounts of follow-up, survival figures and estimates were calculated using the Kaplan-Meier method. Cox regression modeling was used to find predictors of death or transplantation in patients with pure DCM, excluding those with neuromuscular disease and inborn errors of metabolism.

## RESULTS

## Etiology

The majority of children (68%) had viral myocarditis. Familial DCM was found in 20% of children and, very rarely, LV noncompaction (found only in one child). Of the remaining cases of DCM (12%), no identifiable cause was found and they were presumably diagnosed as idiopathic DCM.

For the adult population, the main causes of DCM were CAD in 50%, idiopathic form of DCM in 10%, familial form of DCM in 10%, hypertensive heart disease in 20%, viral myocarditis in 8%, and, rarely, 2% of DCM patients were due to toxic, peripartum cardiomyopathy and LV noncompaction (Fig. 2).

The prognosis was that independent risk factors at DCM diagnosis for subsequent death or transplantation in children cohorts were older age, CHF, lower LV ejection fraction (EF $\leq$ 25%), low global strain, significant mitral valve incompetence, high pressure tricuspid incompetence (pulmonary hypertension), diastolic dysfunction, RV involvement (RVD), and cause of DCM ($p < 0.001$ for all) (Figs. 3 and 4). In adults with DCM, low EF (<30–35%), GLPSS < –7.6%, increased EDV, ESV, LBBB, diastolic dysfunction, RVD, and significant LV dyssynchrony were the main independent risk factors for major cardiac events and need for CRT or transplantation ($p < 0.001$ for all) (Tables 1and 2, Fig. 5) .

## Cohort Differences

The retrospective and prospective cohorts are similar with respect to sex, age, region, cause, presence of CHF at diagnosis, and outcome.

### *Annual Incidence of DCM*

Incidence rates were based on 195 cases of DCM diagnosed from 1996 to 2008 in two regions in the Middle East area (Chest Hospital in Kuwait and Tanta University Hospital in Egypt). The overall rate of pure DCM in childhood was 0.07 cases/100,000/year. The incidence was higher in males than in females (60 and 40%, respectively). The incidence of DCM was significantly higher in adults than children; 10 cases/100,000/year, which could be attributed to the high prevalence of major risk factors for CAD in our communities (smoking, diabetes, hypertension, obesity, dyslipidemia, and family history of CAD) (Fig. 6).

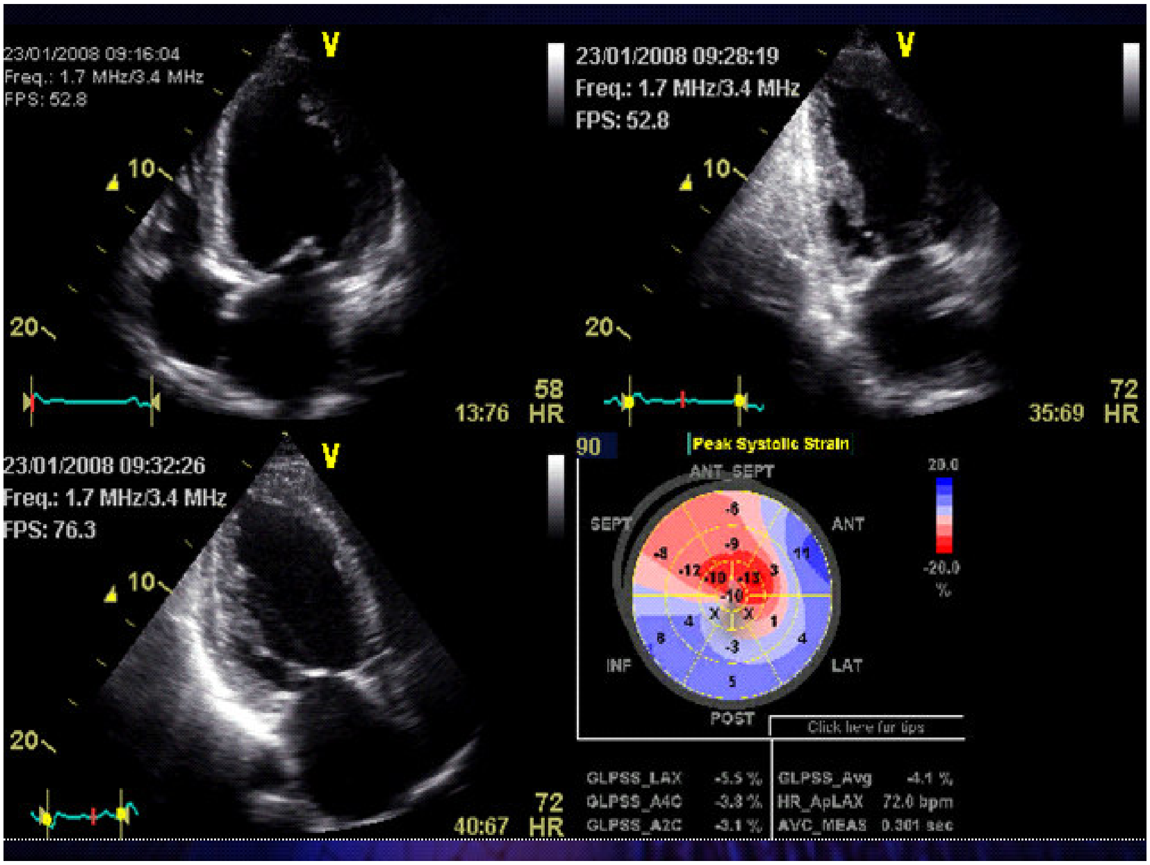

**FIGURE 1**. GLPSS in patients with CAD and severe LV dysfunction, GLPSS = –4.1% , note that tracing of the endocardium through apical four-, two-, and three-chamber views yield the bulls-eye in two-dimensional echocardiography.

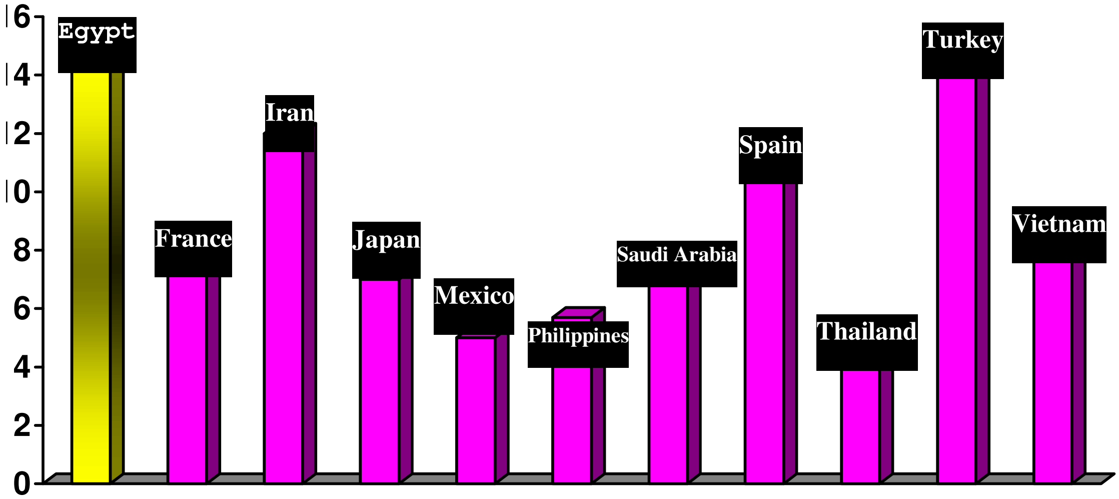

**FIGURE 2**. Incidence of CAD in the Middle East area vs. Europe. Registry by the WHO.

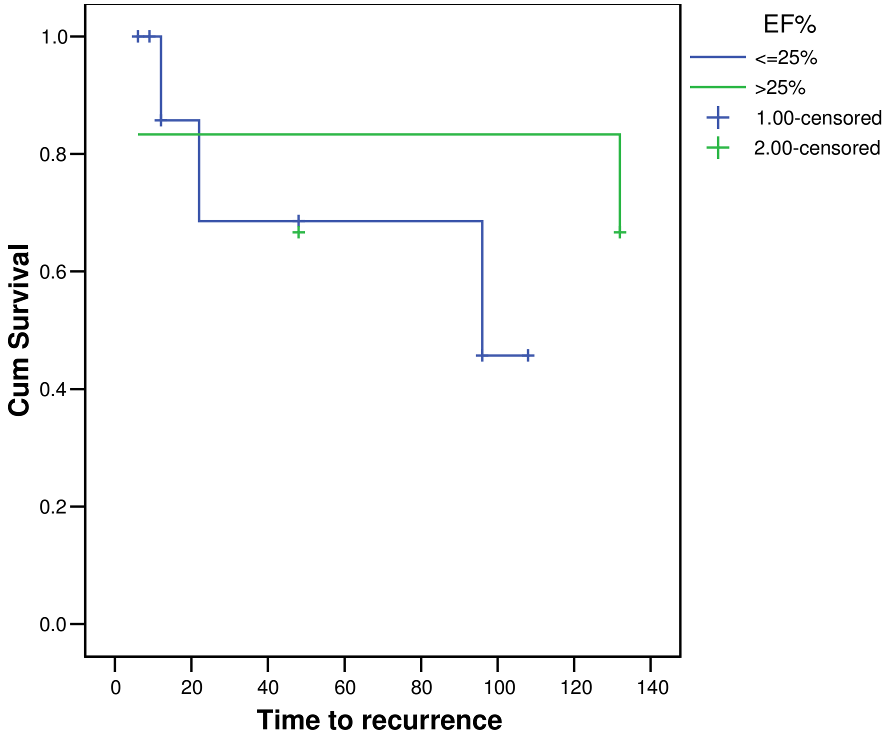

**FIGURE 3.** Freedom from death or transplantation for patients with pure DCM with EF ≤ 25% (GLPSS < –7.6%) and EF > 25%. There was high incidence of recurrence of major cardiovascular events among children with EF < 25% and low two-dimensional strain values.

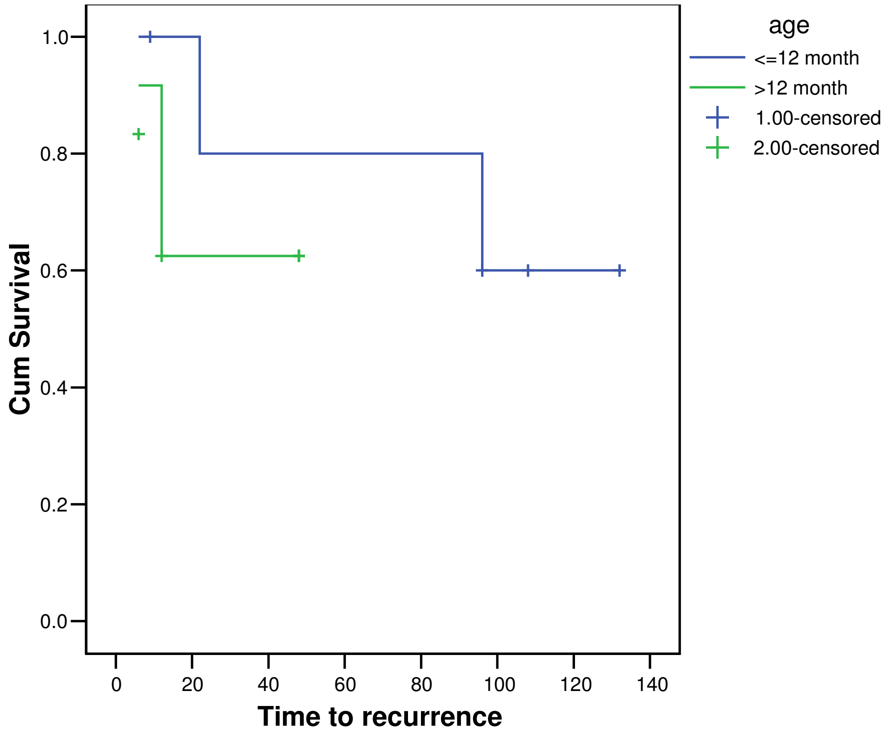

**FIGURE 4.** Kaplan-Meier survival curve of children with DCM (viral, familial, and idiopathic causes). Note the early high rate of death or transplantation at diagnosis.

**TABLE 1**
**Comparison of Measured Variable in Relation to Studied Groups (Adults with DCM vs. Control)**

| Variables | Cases (DCM in Adults) | | | Control | | | t | *p* |
|---|---|---|---|---|---|---|---|---|
| | Range | Mean | SD | Range | Mean | SD | | |
| Age | 20–78 | 48.12 | 12.40 | 18–70 | 44.69 | 12.41 | 1.357 | 0.178 |
| Weight | 45–120 | 80.37 | 16.79 | 42–110 | 79.13 | 16.29 | 0.365 | 0.716 |
| EF% | 12–50 | 34.09 | 9.81 | 45–76 | 60.93 | 7.94 | 14.668 | 0.001* |
| AFI (GLPSS) | 0–18 | 8.67 | 3.71 | 11–25 | 17.48 | 3.34 | 12.200 | 0.001* |
| EDV | 82–365 | 164.24 | 57.22 | 51–220 | 94.82 | 32.07 | 6.772 | 0.001* |
| ESV | 45–388 | 110.23 | 61.25 | 14–71 | 37.93 | 15.12 | 7.277 | 0.001* |
| IVS | 3–24 | 10.12 | 3.34 | 6–16 | 11.28 | 3.39 | 1.769 | 0.081 |
| PWT | 7–17 | 10.67 | 2.29 | 6–15 | 10.89 | 2.16 | 0.438 | 0.662 |

* Significant.

**TABLE 2**
**Correlation between EF% and GLPSS**

| GLPSS | EF% | |
|---|---|---|
| | r | *p* |
| Cases | 0.719 | 0.001* |
| Total control | 0.744 | 0.001* |
| Control with normal EF% | 0.593 | 0.001* |
| Control with abnormal EF% | 0.687 | 0.019* |

* Significant.

## Clinical Presentation

Clinical findings, therapy, and outcomes are based on the entire cohort of 195 patients with pure DCM, unless otherwise specified. The median age at diagnosis was 2.5 years for children and 48.1 ± 12.4 years for adults (Table 1). In the children's group, age younger than 1 year was the most common age at diagnosis of DCM (60%). The 6- to 18-year-old age group was the least common (10%) age at initial diagnosis. The majority of children had clinical evidence of CHF at diagnosis (90%), with 60% overall classified as having class IV heart failure with EF ≤ 25%. In adults with DCM, advanced symptoms of heart failure (NYHA class III-IV), pulmonary edema, ventricular tachyarrhythmia, and cardiac arrest correlated with EF < 30–35% and low GLPSS (< –7.1 ± 0.5%) at high sensitivity (90%) and specificity (91%). At this cutoff value (GLPSS < –7.1 ± 0.5 %), we found that major cardiac events frequently occurred (23 out of 30 patients developed pulmonary edema, need for inotropic infusion, sudden death, frequent rehospitalization for control of CHF symptoms, need for CRT and cardiac transplantation). In comparison, patients with GLPSS > –7.6% had very low incidence of major cardiac events (only six patients out of 75 patients developed major cardiac events) (Fig. 3).

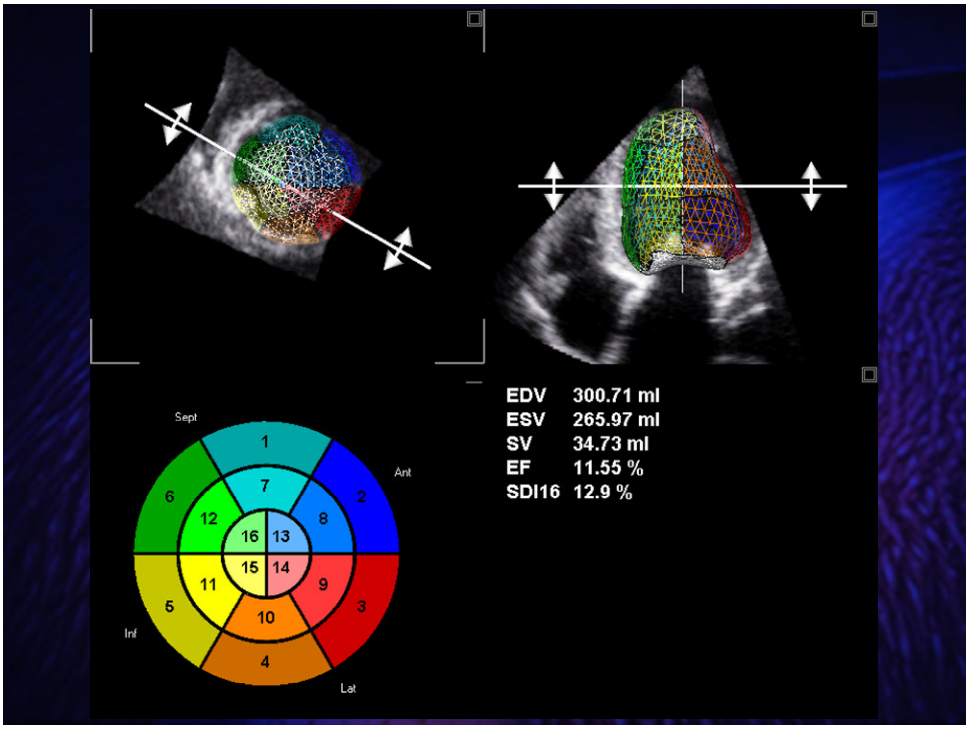

**FIGURE 5.** Young adult with severe form of DCM, his EF = 11.9% only, measured by three- and four-dimensional LV volume data set. Note the evidence of significant LV intraventricular mechanical dyssynchrony; SDI (systolic dyssynchrony index) = 12.9%.

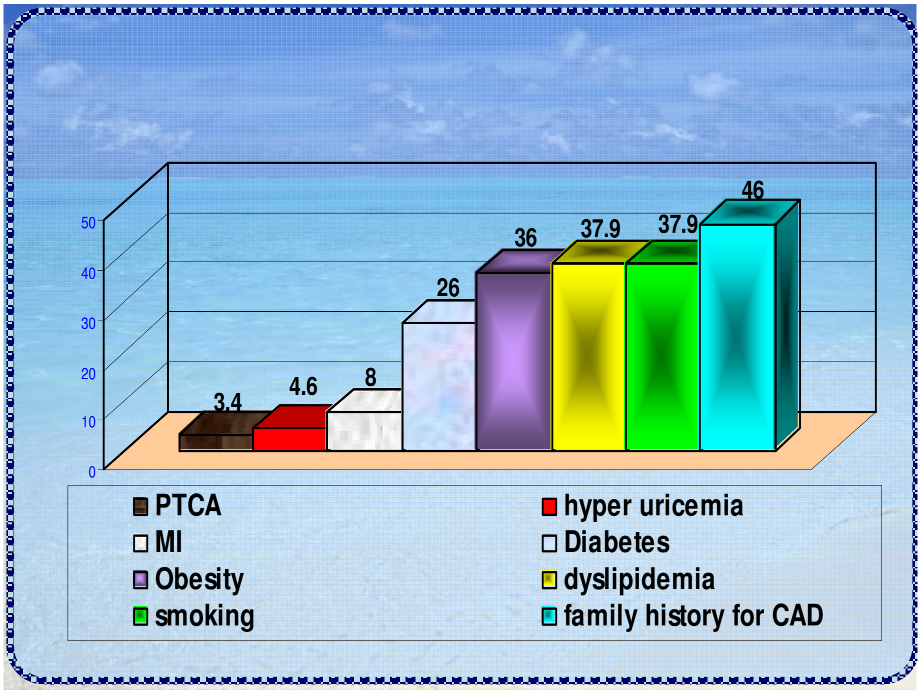

**FIGURE 6.** The percentage of the most currently associated risk factors among hypertensive Egyptians who presented with chest pain for evaluation in Alpha Center, Cairo, ARE. (From Elkilany, G.E.N. [2005] Myocardial perfusion imaging of high risk hypertensive population in Egypt. *Egypt. J. Hypertens. Cardiovasc. Risk* **1(2),** 32–38. Presentation during the World Hypertension League 2005 Meeting.)

## Detailed Echocradiographic Criteria

Echocardiogram results were available for most of the children (70%) and all adult patients. The mean LVEDV and LVESV in adults was 164 ± 57 and 110 ± 61 ml, respectively, whereas the mean LVEDD was 5 ± 0.6 cm and LVESD was 3.5 ± 0.5 cm in children. LV ejection fraction was severely depressed (34 ± 9.8%) and GLPSS was markedly blunted (–8.7 ± 3.7%) in DCM patients in both groups in comparison with control subjects (61 ± 7.9% and –17.5 ± 3.3% for EF and GLPSS, respectively; $p <$ 0.001)(Fig. 7). LV end-diastolic posterior wall thickness and septal wall thickness were, on average, normal, but LV mass was mildly abnormal in children. In adults, LV hypertrophy (LVH) was found in 24 patients with variable grades of diastolic dysfunction, whereas significant LV intraventricular mechanical dyssynchrony (SDI > 10 by four-dimensional LV volume) was evident in 17 adult patients (EF ranges from 11.8 to 30% and GLPSS ranges from –4 to –7.8%), which was associated with advanced CHF symptoms (NYHA functional class IV, pulmonary edema, paroxysmal nocturnal dyspnea).

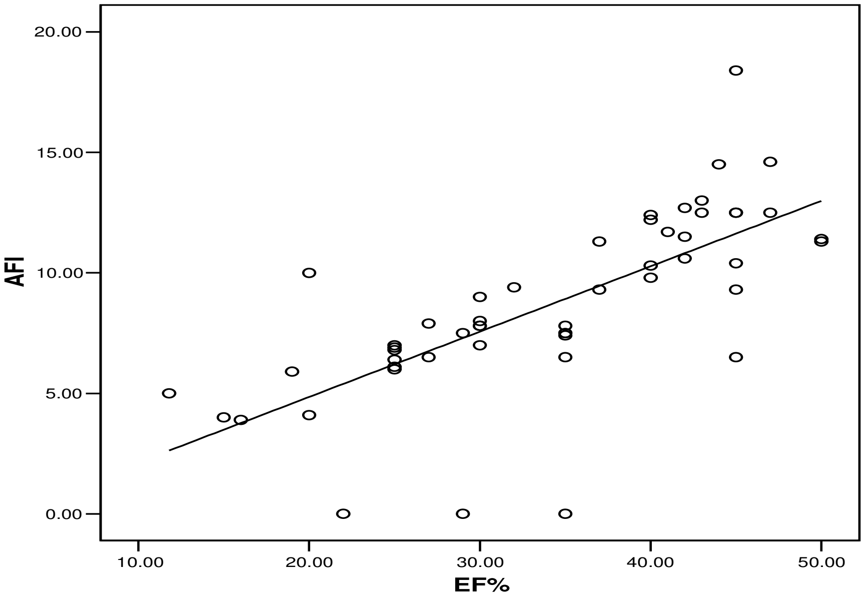

**FIGURE 7.** Correlation between EF% and AFI (GLPSS) among DCM patients.

## *Therapy*

At the time of diagnosis of DCM, 80% of patients were prescribed loop diuretics and digitalis, and 60% an angiotensin-converting enzyme inhibitor, with 20% receiving an antiarrhythmic agent (mainly amiodarone). L-Carnitine, aldosterone antagonism, gamma globulins, and aspirin were prescribed routinely for most of the children with DCM at the Chest and Tanta University Hospitals. Antithrombotic therapy was prescribed for children with very low ejection fraction (EF < 20%) and inotropes in acute heart failure. There was low use of beta-blockers in young children (beta-blockers were not given in acute setting at our center, they were prescribed only after 3 months in children if ejection fraction remained severely depressed [<20%], or the child remained symptomatic despite maximal medical therapy). Pacemaker and balloon pump use at diagnosis was rare (1% each), as was use of biventricular pacing (2%) and (15%), in children and adults, respectively.

Interestingly, Carvedilol saved four lives in children (who were rehospitalized for recurrence of symptoms of CHF with significant drop of EF < 30%) when added to ACE inhibitors, digitalis, and diuretics regimen; EF improved significantly from 20–25% to 45–50% with dramatic improvement of symptoms and clinical status on follow-up (for 2 years). Two of them had viral etiology of DCM (2 years old), one had idiopathic DCM (4 years old), and the last one was a known case of familial DCM (9 years old).

## Clinical Outcomes

The median age of the patients at the time of diagnosis was 2.5 and 48 years for children and adults, respectively. There was an early high rate of death or transplantation in children after diagnosis (Fig. 4). Although the initial cure rate of viral myocarditis-induced DCM in children was high, one-third of them developed recurrence of CHF symptoms with severe depression of LV systolic function on follow-up. In adults, the median age of death in DCM patients (ischemic LV dysfunction included) was 51 years, which is concordant to an INTERHEART of study by S. Yusuf published in *The Lancet* 2004.

Kaplan-Meier analysis of survival after DCM diagnosis revealed 1-year survival of 92% for children (Fig. 8).

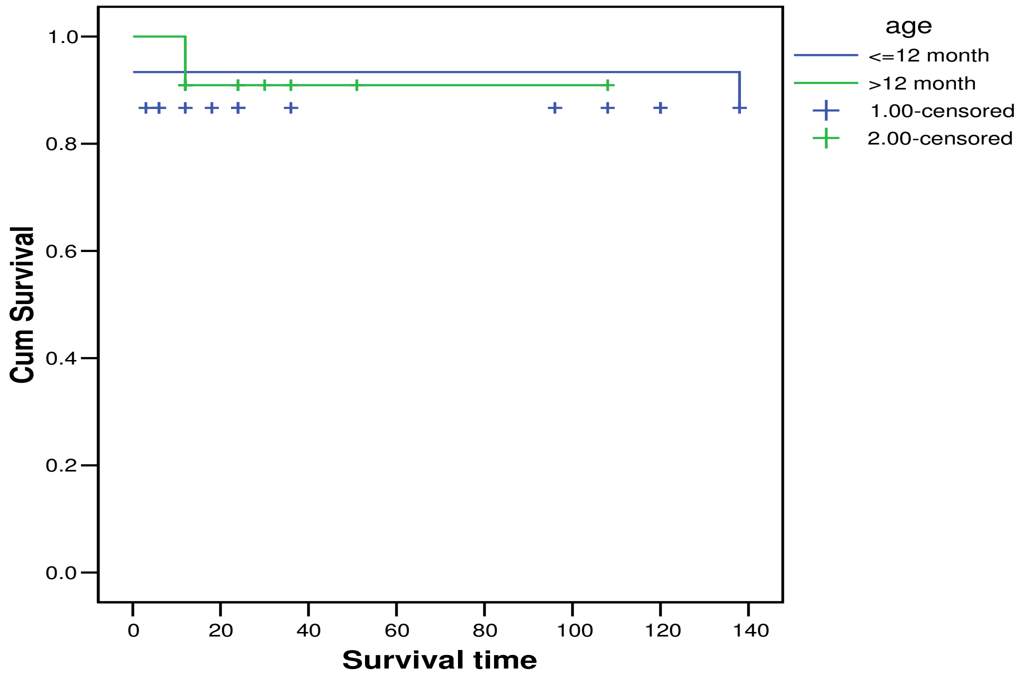

**FIGURE 8.** Kaplan-Meier analysis of survival after DCM diagnosis. Included different causes of DCM in children: vira , familial, and idiopathic forms of DCM. (Copyright reserved for the author: Dr. Galal Eldin Nagib Elkilany.)

In all adults as well as in 70% of children cohorts, a detailed echocardiographic examination (by two- and three-dimensional echocardiography) was done and revealed that certain parameters were independent predictors of death, recurrence of CHF symptoms, deterioration, and need for cardiac transplantation or CRT:

- Low ejection fraction (Fs), of moderate severity or more; EF < 30–35% for adults and ≤25% for children (Fig. 3)
- Pulmonary hypertension, PASP > 40–45 mmHG for children only

- Significant mitral incompetence; moderate or more (grade II–IV/IV) for both groups
- Diastolic dysfunction of LV of moderate or more (grade II–IV) for both groups
- Low GLPSS, < –7.1 ± 0.5% for adults and children (Figs. 3 and 7)
- RV impairment, for both groups
- Significant LV dyssynchrony and increased EDV, ESV by four-dimensional LV volume (for adults only with DCM) (Fig. 5)
- LBBB, for adults only, $p < 0.001$ for all

## Predictors of Clinical Outcome

- **In Children** — Risk factors for the composite end point of death, transplantation, and need for CRT were age at diagnosis, cause, CHF at diagnosis, and ejection fraction, GLPSS, EDD, ESD, mitral incompetence, diastolic dysfunction, and RV involvement ($p < 0.001$ for all). Children with idiopathic disease had a twofold worse outcome than those with myocarditis. A higher fractional shortening (EF > 25%) and global strain was associated with better outcome (Figs. 3 and 4).
- **In Both Groups (Children and Adults)** — All echocardiographic measures examined were univariately associated with DCM outcome except for septal and posterior wall thickness, but ejection fraction (EF) (which showed excellent correlation with GLPSS, r = 0.719) was the only independent echocardiographic risk factor, (Tables 1 and 2, Fig. 7).

A cutoff value for GLPSS of –7.1 ± 0.5% had 90% sensitivity and 91% specificity for detection of patients with severe LV systolic dysfunction (EF ≤ 30–35%), and patients at risk for major cardiac events (cardiac death, acute heart failure, pulmonary edema, frequent hospitalization for progressive heart failure symptoms, need for CRT, and transfer for cardiac transplantation). In short-term follow-up (140 days), 23 patients out of 30 (who had GLPSS of –7.6% or less) developed major cardiac events (acute heart failure, pulmonary edema, ventricular tachyarrhythmia, cardiac arrest, cardiogenic shock, and need for CRT or cardiac transplantation).

## DISCUSSION

DCM in children and adults is a diverse disorder with outcomes that depend on cause and age at presentation, as well as heart failure status and certain echocardiographic criteria of risk. Early failure of medical management, high mortality rates, and progressive deterioration are found regardless of etiology[3].

The universal incidence of DCM in children was 0.56 cases/100,000/year, tenfold lower than in adults[21,22]. This may relate to fewer chronic health habit–associated risk factors, a longer latency period for clinical expression of the effects of genetic and environmental factors on the heart, and the wider age span of adulthood compared with childhood, giving adults more opportunity to develop DCM. When study differences are accounted for, the incidence of pediatric DCM in the U.S. is similar to that reported in Finland (0.65/100,000 aged ≤20 years) and, after accounting for age, Australia (1.09/100,000 aged ≤10 years)[20,21,22]. Boys have a higher DCM incidence than girls, related to X-linked genetic causes and neuromuscular disorders. Black children have higher rates of DCM and different causes of DCM than do white children.

DCM is significantly more likely to present in the first year of life than at older pediatric ages. Infants had very high incidence of DCM than older children. However, DCM presenting at older pediatric ages is, in general, associated with worse outcomes. In addition to older age, worse ventricular dysfunction at presentation and more advanced CHF at presentation were associated with worse outcomes (Figs. 3 and 4).

The cause of DCM was an independent predictor of the composite outcome of death or transplantation. Outcomes by cause varied widely from 57 to 94% survival at 5 years, suggesting the need to establish an etiology to determine the subsequent optimal management and to more accurately predict prognosis. However, understanding the cause of DCM remains difficult, with only 30% of pediatric patients having an identifiable cause in different studies. The spectrum of disease etiologies in childhood is quite different than that reported in adults. In adults, CAD is a common cause of DCM, which is very rare in childhood, and explains the differences between incidence rates in childhood vs. adulthood.

Mortality and cardiac transplantation rates did not match for specific causes of pediatric DCM. There was lower mortality, but a higher rate of cardiac transplantation for familial DCM compared with idiopathic DCM. Idiopathic DCM had high rates of both death and cardiac transplantation. This raises questions about optimal cardiac transplantation management. One conclusion would be that patients with idiopathic DCM do not undergo transplantation as often as they should, since mortality remains elevated, or that more needs to be done to establish etiologies for idiopathic DCM in pediatric patients. Similar questions are raised for myocarditis, in which deaths continue to occur years after presentation. The continuing mortality risk contradicts the previously held belief of a high recovery rate in this population. Familial DCM has high early transplantation rates and lower mortality compared with other causes, suggesting that families and their care providers may be more prepared to allow transplantation in these young patients early[1].

There are limitations to this study. First, subclinical cases of DCM are, by definition, not completely captured by the methods used in this study. For this reason, the incidence of DCM is probably underestimated and disease severity is possibly overestimated. In addition, there was a lack of definite diagnosis of certain types of DCM (idiopathic and familial) enrolled in this study. Finally, detailed treatment data only collected from the retrospective cohort preclude reliable conclusions regarding potential associations between therapy and outcomes in this cohort. However, therapies have not been shown to affect outcomes dramatically[21,24].

Despite the billions of dollars used to care for these patients, develop new therapies, and perform genetics-based studies, survival is still poor. New methods for early diagnosis[25] and risk stratification, as well as new therapies, need to be developed for infants and children with DCM to avoid transplantation and premature death[3,26]. Familial DCM had the best survival (96%) at 1 year after diagnosis in the present study (similar to that found by Towbin et al.[27]). Unfortunately, half of those patients (who showed initial improvement) developed recurrence of symptoms of CHF and severe deterioration of LV function (Fs < 10%) on follow-up (Figs. 3 and 4). Although patients with viral myocarditis had excellent outcomes (92% survival at 1 year), one-third of those children (who initially presented with EF < 25%) showed deterioration on follow-up with recurrent symptoms of severe CHF and marked impairment of LV systolic function indices. Based on our multivariable model and the Towbin et al. publication[27], a patient presenting with DCM and a significant degree of LV dysfunction with a fractional shortening <20% (EF < 40%) has a risk of death or cardiac transplantation increased by 2.2 times (hazard ratio, 2.19; 95% confidence interval, 1.55–3.08) compared with a patient with better LV function and GLPSS.

## CONCLUSION

New methods for early diagnosis and risk stratification, as well as new therapies, need to be developed and standardized for children and adults with DCM to avoid transplantation and premature death. We found that early and definite diagnosis by two- and three-dimensional echocardiography with combined use of global strain (AFI) could be of great help for risk stratification of such patient populations. It is of crucial importance to include other important detailed echocardiographic parameters in risk-stratifying patients with DCM — RV function, diastolic function, mitral valve function, pulmonary artery pressure, LBBB, and degree of LV dyssynchrony — in order to optimize the best therapeutic strategy for DCM patients. Screening of adult populations who are at risk of developing CAD and LV dysfunction is a must to reduce the burden of DCM; early initiation of preventive measures and effective therapeutic agents of such patient populations could reduce incidence of CAD, which constitutes 50% of adult patients with DCM. In addition, the effective therapeutic agents (ACE inhibitors, beta-blockers, and biventricular pacing) that have been used frequently in adults are still underutilized in the children's group. A lot of work must be done to identify at which age we should start beta-blocker therapy and biventricular pacing.
